# Supplementary material for: One-dimensional organic lead halide perovskites with efficient bluish white-light emission
Source: Nat Commun. 2017 Jan 4;8:14051. doi: 10.1038/ncomms14051 (PMC5216108; doi:10.1038/ncomms14051)
Supplement: Supplementary Information — Supplementary Figures and Supplementary Tables [file ncomms14051-s1.pdf]

Supplementary Table 1 | Single crystal X-ray diffraction data of 1D bulk single crystal perovskites.

Empirical formula	C4 H14 Br4 N2 Pb	
Formula weight	617.00	
Temperature	103(2) K	
Wavelength	0.71073 Å	
Crystal system	Orthorhombic	
Space group	I m m a	
Unit cell dimensions	a = 14.6230(16) Å	α= 90°.
	b = 6.1041(7) Å	β= 90°.
	c = 14.4070(16) Å	γ = 90°.
Volume	1286.0(2) $Å^3$	
Z	4	
Density (calculated)	3.187 Mg/m^3	
Absorption coefficient	25.504 mm^{-1}	
F(000)	1096	
Crystal size	0.280 x 0.095 x 0.060 mm^3	
Theta range for data collection	1.984 to 28.488°.	
Index ranges	-18<=h<=19, -7<=k<=8, -19<=l<=19	
Reflections collected	7142	
Independent reflections	895 [R(int) = 0.0481]	
Completeness to theta = 25.242°	99.9 %	
Absorption correction	Empirical	
Max. and min. transmission	0.2163 and 0.07491	
Refinement method	Full-matrix least-squares on F^2	
Data / restraints / parameters	895 / 0 / 39	
Goodness-of-fit on F^2	1.137	
Final R indices [I>2sigma(I)]	R1 = 0.0182, wR2 = 0.0416	
R indices (all data)	R1 = 0.0185, wR2 = 0.0417	
Extinction coefficient	0.00168(9)	
Largest diff. peak and hole	1.068 and -1.161 e.$Å^{-3}$	

Supplementary Table 2 | Chemical yield and composition of the component molar ratio in 1D microscale perovskites according to TGA and ^{1}H NMR data.

Product	Reagent	Starting amount	Molar ratio[a]	Theoretical ratio[b]	Yield
	Octanoic Acid	1.00 mmol	0.00	0.00	
Microscale Perovskites	N, N'-dimethylethylenediamine	0.30 mmol	1.02	1.00	95 %
	$PbBr_2$	0.10 mmol	1.00	1.00	

[a] Based on ^{1}H NMR and TGA data; [b] based on the chemical formula: $C_4N_2H_{14}PbBr_4$.

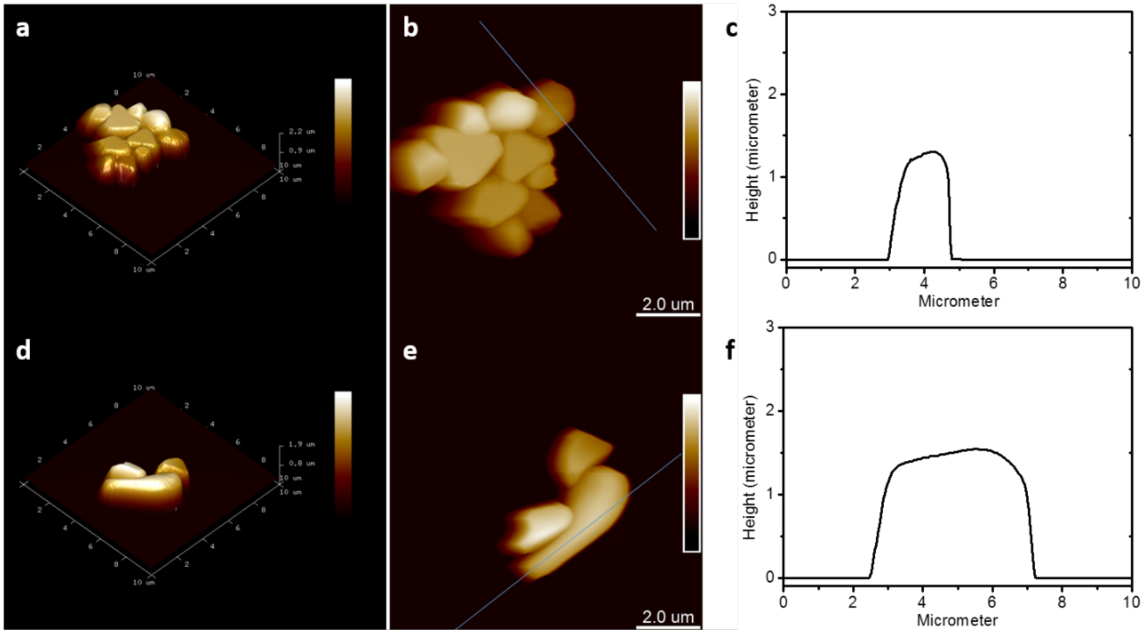

Supplementary Figure 1 | AFM characterization of the 1D microscale perovskites. **a,d,** 3D AFM images of $C_4N_2H_{14}PbBr_4$ microscale perovskites. **b,e,** 2D AFM images of the as-prepared microscale perovskites. **c,f,** Height profile along the blue line in 2D AFM images (b) and (e).

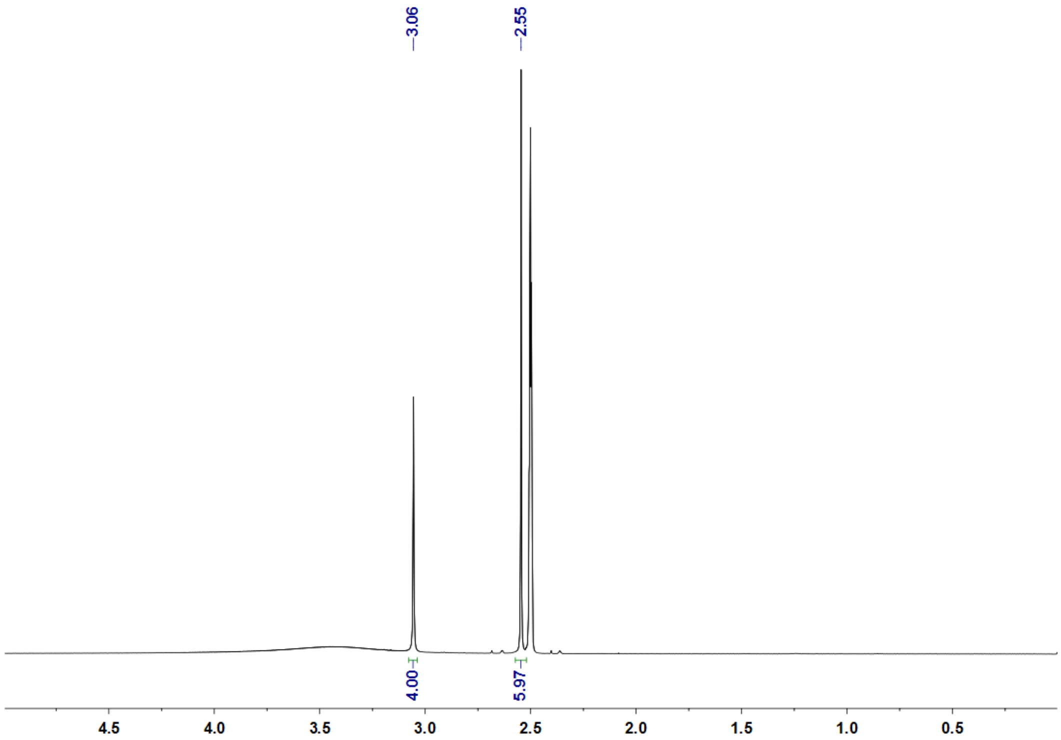

Supplementary Figure 2 | Proton NMR spectrum of the 1D microscale perovskites in DMSO-d$_6$.

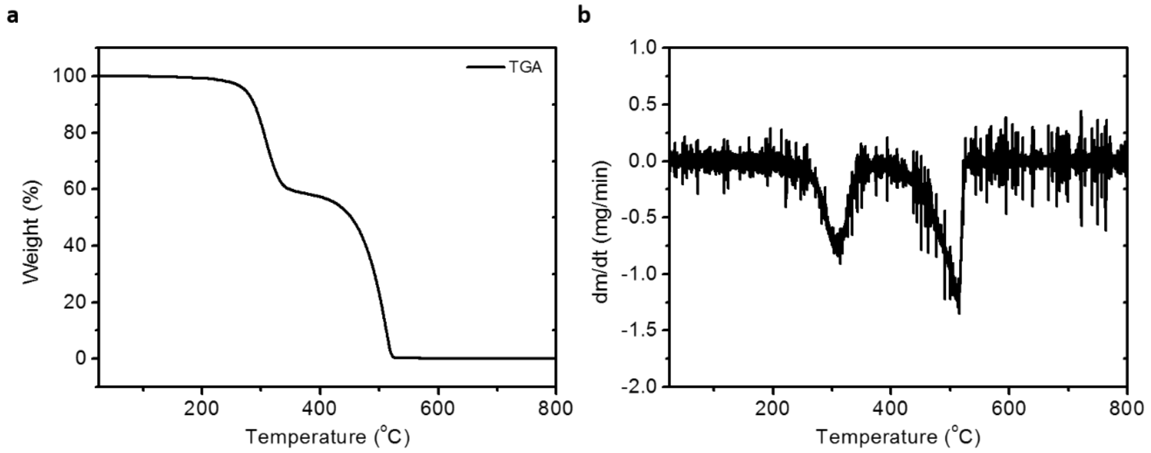

Supplementary Figure 3 | TGA study of the 1D microscale perovskites. a, TGA heating curves of $C_4N_2H_{14}PbBr_4$ microscale perovskites. **b,** The corresponding 1st derivative.

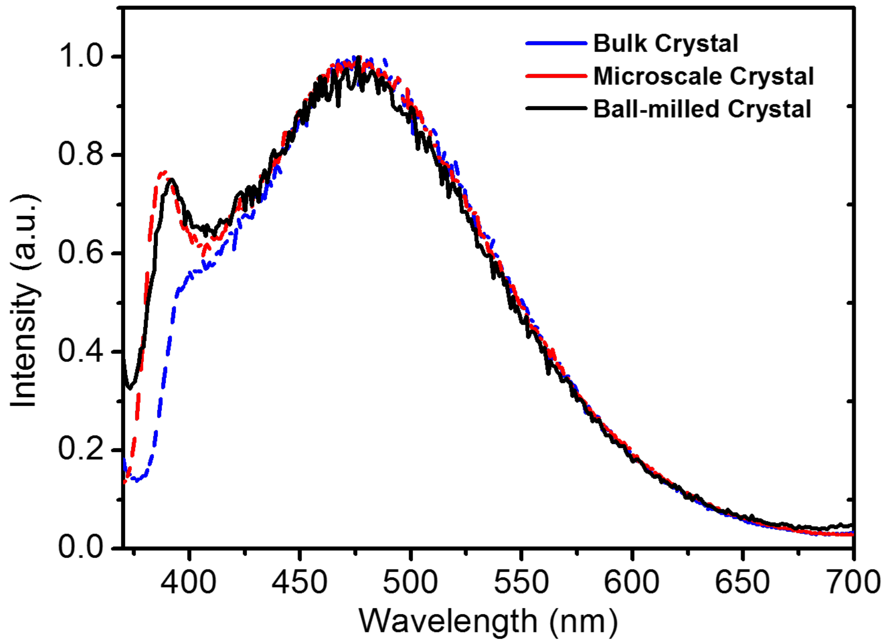

Supplementary Figure 4 | Emission (excited at 360 nm) spectra of ball-milled bulk crystals, as well as bulk and microscale crystals at room temperature.

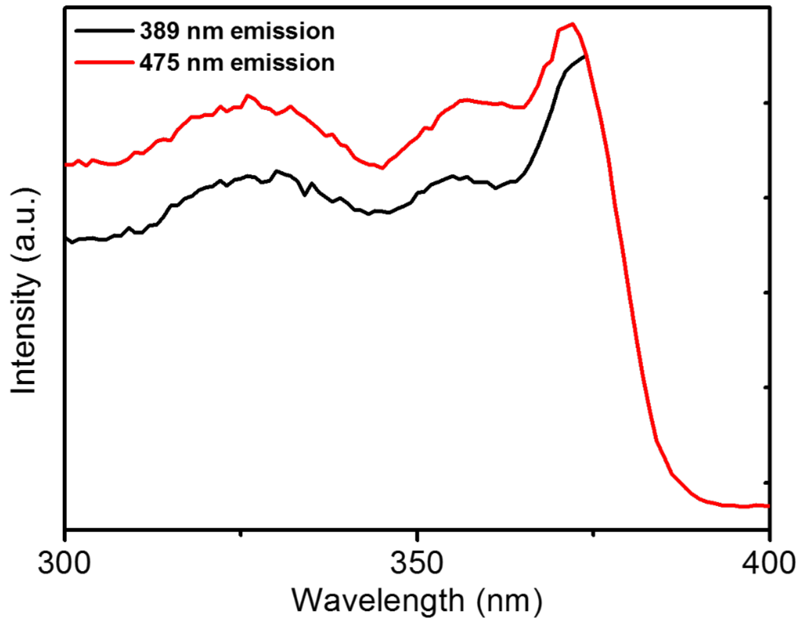

Supplementary Figure 5 | Excitation spectra for the 389 and 475 nm emissions of the 1D microscale perovskites.

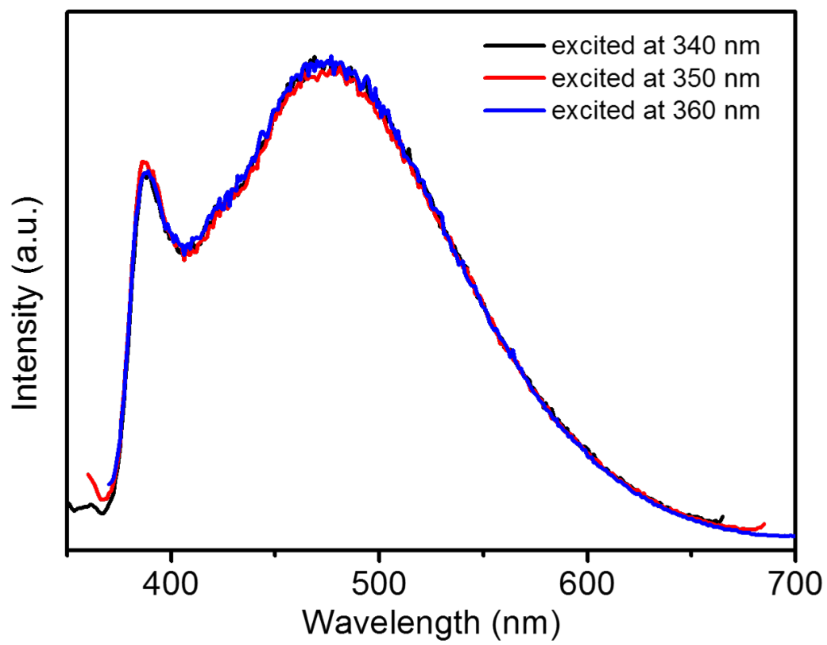

Supplementary Figure 6 | Emission spectra of the 1D microscale perovskites at different excitation wavelengths.

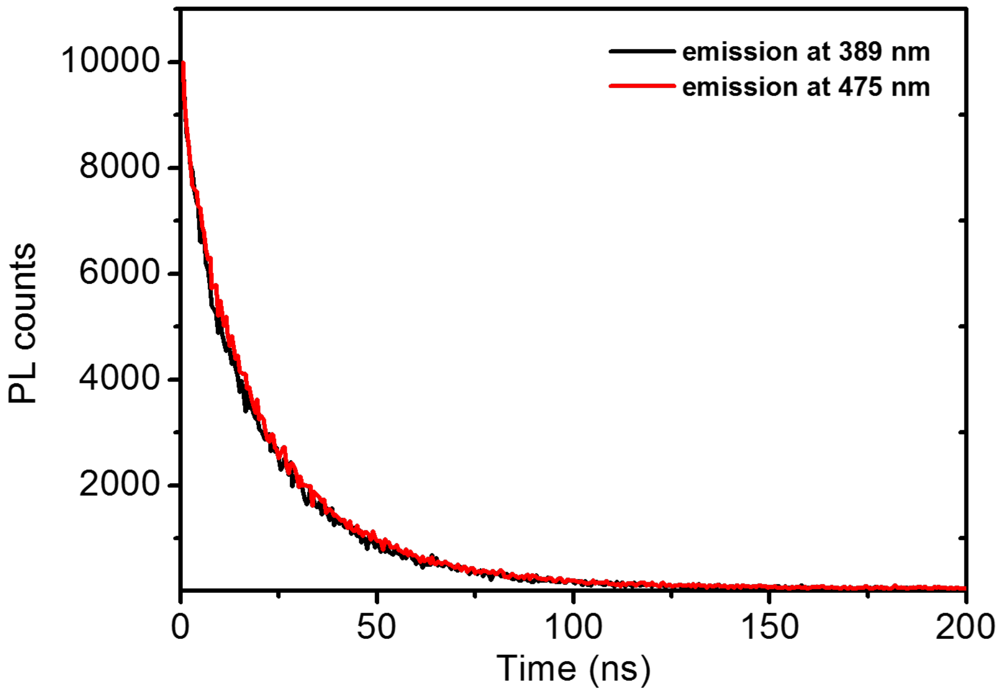

Supplementary Figure 7 | Decay curves monitored at 389 nm and 475 nm for microscale crystals at room temperature.

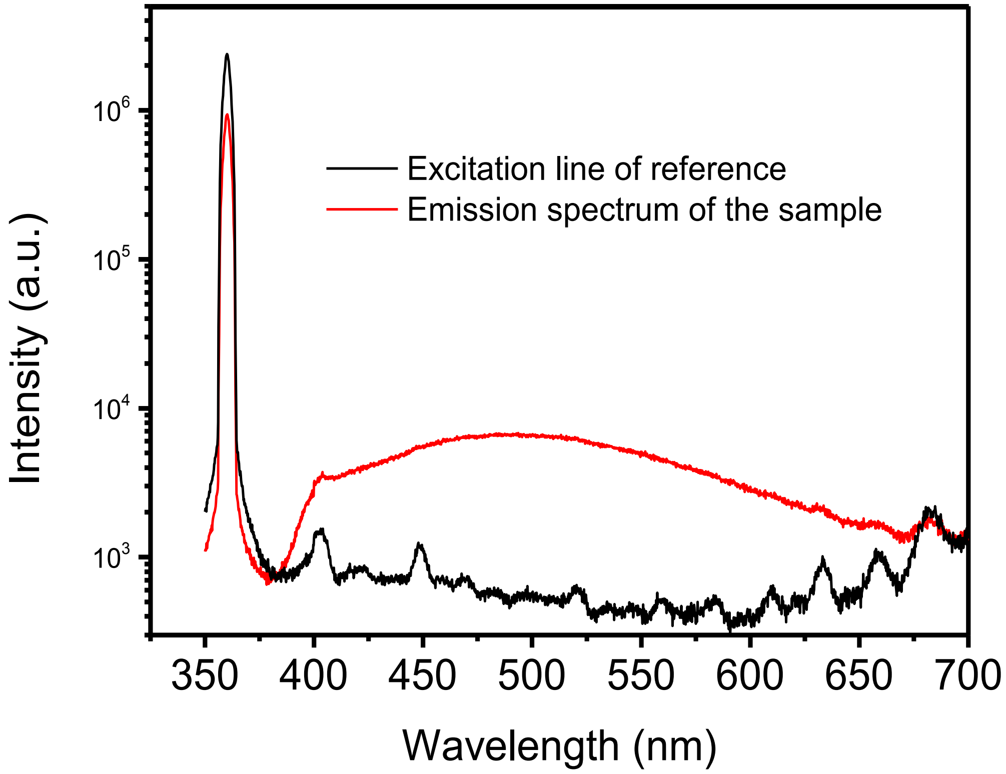

Supplementary Figure 8 | Excitation line of reference and emission spectrum of 1D Pb bromide perovskite bulk crystals by an integrating sphere.

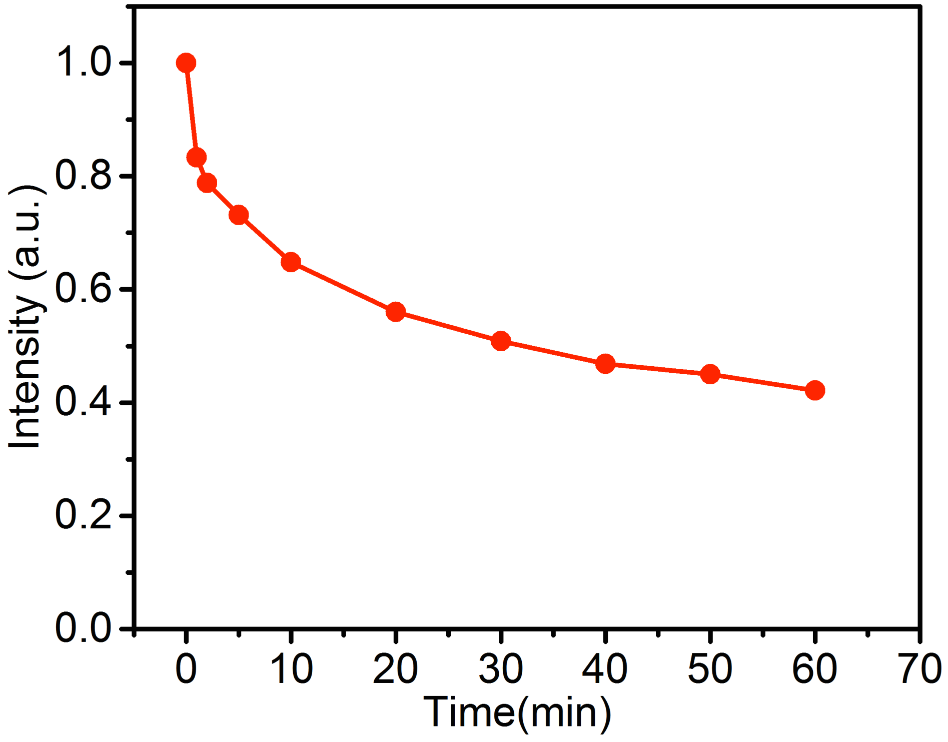

Supplementary Figure 9 | Photoluminescence intensity changes after continuous illumination in air using Hg lamp.

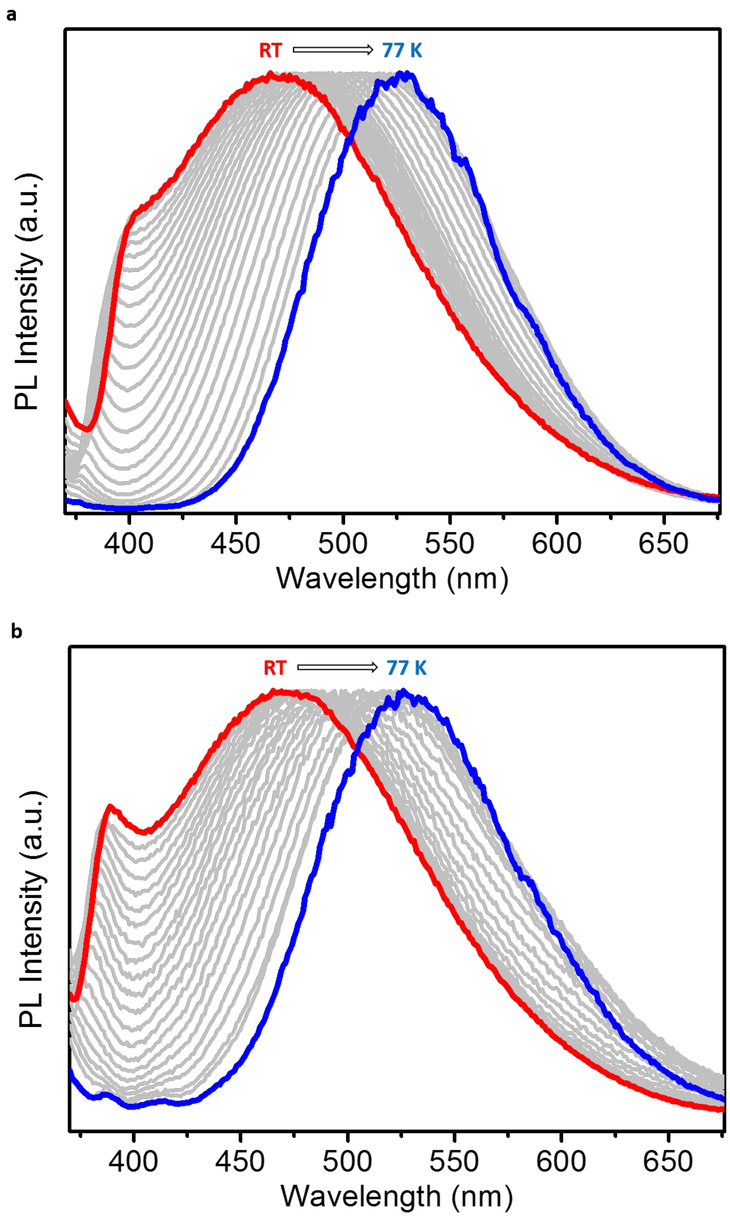

Supplementary Figure 10 | Temperature dependent emission spectra of bulk (a) and microscale (b) crystals from room temperature to 77 K (one scan per 10 K, spectra are normalized for clarity).
